# Trends of risk classification and primary therapy for Japanese patients with prostate cancer in Nara Uro-Oncological Research Group (NUORG)–a comparison between 2004-2006 and 2007-2009

**DOI:** 10.1186/1471-2407-13-588

**Published:** 2013-12-10

**Authors:** Nobumichi Tanaka, Akihide Hirayama, Tatsuo Yoneda, Katsunori Yoshida, Keiji Shimada, Noboru Konishi, Kiyohide Fujimoto

**Affiliations:** 1 Department of Urology, Nara Medical University, Nara, Japan; 2 Department of Pathology, Nara Medical University, Nara, Japan

**Keywords:** Primary therapy, Primary androgen deprivation therapy, Radical prostatectomy, Radiation therapy, Risk classification, Active surveillance

## Abstract

**Background:**

To assess the trends of risk classification and primary therapy in Japanese patients who were diagnosed with prostate cancer between 2004-2006 and 2007-2009.

**Methods:**

A total of 4752 patients who were newly diagnosed with prostate cancer at Nara Medical University and its 23 affiliated hospitals between 2004 and 2009 were enrolled. The differences in risk classification and primary therapy were compared in patients who were newly diagnosed between 2004-2006 (prior period) and 2007-2009 (latter period).

**Results:**

The proportion of patients with a high or greater risk significantly decreased in the latter period compared to the prior period (p < 0.001). The proportion of primary androgen deprivation therapy (PADT) was 50% in the prior period, and 40% in the latter period. On the other hand, the proportion of radiation therapy was 14% in the prior period, but 24% in the latter period. The proportion of radical prostatectomy was the same in the two periods (30%). The primary therapy was significantly different between the two periods (p < 0.001).

**Conclusions:**

Higher risk patients significantly decreased in the latter period compared to the prior period. The use of PADT also significantly decreased in the latter period. However, there were still higher risk patients in Japan, and the use of PADT was still common in patients with localized prostate cancer or locally advanced prostate cancer in Japan.

## Background

There is a distinctive trend in Japan that a large proportion of patients who are diagnosed with prostate cancer choose primary androgen deprivation therapy (PADT) as the primary therapy. We have previously reported that there is a significant difference in the risk classification and primary therapy between Japanese and USA patients [1]. The proportion of high risk patients was significantly higher in Japan than in the USA, and the proportion of patients undergoing PADT was also significantly higher in Japan than the USA [2-4]. Following our first report, we have conducted a further investigation between 2007 and 2009.

In this paper, we report the trend of risk classification and primary therapy in patients who were diagnosed with prostate cancer between 2007 and 2009 in the Nara Uro-Oncological Research Group (NUORG) registry, and compare the results with those of the previous survey performed between 2004 and 2006.

## Methods

A total of 4752 patients who were newly diagnosed with prostate cancer at Nara Medical University hospital and its 23 affiliated hospitals between January 2004 and December 2009 were enrolled in this retrospective study. The clinical TNM classification (UICC 2002), biopsy Gleason score, PSA at diagnosis and primary therapy were surveyed. We used the risk classification reported by D’Amico [5]. Patients with cT3-4N0N0 were further defined as “very high” risk, and patients with node metastasis or distant metastasis were defined as “metastatic.”

We compared the baseline characteristics (stage, PSA distribution, age, Gleason score, and risk classification) between the prior (2004-2006) and latter (2007-2009) periods. Any differences in the primary therapy between the prior and latter periods were also compared.

To examine the differences in categorical parameters, the chi-square test was performed. The Mann–Whitney U test was used to compare metric variables. All statistical analyses were performed using PASW Statistics 17.0 (SPSS Inc., Chicago, IL, USA). All p values < 0.05 were considered to be statistically significant.

The Medical Ethics Committee of Nara Medical University approved this retrospective study, and it was exempted to obtain informed consent from the patients in consideration of the aim and methods of this study.

## Results

The demographic characteristics of all 4752 patients are shown in Table 1. The mean (median) values of patients’ age were 71.8 (72.0) and 71.9 (72.0) years in the prior and latter periods, respectively. The mean (median) values of the PSA value at the time of diagnosis in the prior and latter periods were 137.9 (12.2) and 102.1 (10.8) ng/mL, respectively. There was a significant difference in the PSA value at diagnosis between the prior and the latter periods (p = 0.025, Mann–Whitney U test). The proportions of older patients and those with a higher PSA value at the time of diagnosis were significantly higher in the prior than the latter period (p = 0.036 and p < 0.001). On the other hand, the proportion of Gleason 7 was significantly higher in the prior than the latter period (p < 0.001). There were no differences in the clinical T and N stage distribution between the two groups, while the proportion of metastatic patients was significantly higher in the prior than the latter period (p = 0.008). Regarding risk classification, the proportion of high risk patients was significantly higher in the prior than the latter period (p < 0.001).

**Table 1:** Demographic characteristics of 4752 patients

	**Overall**	**2004-06**	**2007-09**	***P *** **value**
	**n = 4752 (%)**	**n = 2303 (%)**	**n = 2449 (%)**	
*Age (year)*				
Younger than 60	278 (5.9)	154 (6.7)	124 (5.1)	
60-69	1423 (29.9)	68.4 (29.7)	739 (30.2)	
70-79	2367 (49.8)	1117 (48.5)	1250 (51.0)	
80 or older	684 (14.4)	348 (15.1)	336 (13.7)	0.036
*PSA at diagnosis*				
10.0 or less	2123 (44.7)	963 (41.8)	1160 (47.4)	
10.1-20	1117 (23.5)	554 (24.1)	563 (23.0)	<0.001
Greater than 20	1512 (31.8)	786 (34.1)	726 (29.6)	
*Gleason score*				
2-6	1771 (37.3)	906 (39.3)	865 (35.3)	
7	1614 (34.0)	722 (31.4)	892 (36.4)	
8-10	1367 (28.8)	675 (29.3)	692 (28.3)	0.001
*Clinical T stage*				
T1	1605 (33.8)	766 (33.3)	839 (34.3)	
T2	1919 (40.4)	933 (40.5)	986 (40.3)	
T3	978 (20.6)	489 (21.2)	489 (20.0)	
T4	250 (5.3)	115 (5.0)	135 (5.5)	0.593
*Clinical N stage*				
N0	4439 (93.4)	2161 (93.8)	2278 (93.0)	
N1	313 (6.6)	142 (6.2)	171 (7.0)	0.141
*Clinical M stage*				
M0	4226 (88.9)	2019 (87.7)	2207 (90.1)	
M1a	17 (0.4)	11 (0.5)	6 (0.2)	
M1b	468 (9.8)	257 (11.2)	211 (8.6)	
M1c	41 (0.9)	16 (0.7)	25 (1.0)	0.008
*Risk classification*				
Low	988 (20.8)	474 (20.6)	514 (21.0)	
Intermediate	1252 (26.3)	561 (24.4)	691 (28.2)	
High	1232 (25.9)	626 (27.2)	606 (24.7)	
Very high	657 (13.8)	319 (13.9)	338 (13.8)	
Metastatic	623 (13.1)	323 (14.0)	300 (12.3)	<0.001

### Differences in primary therapy

**Figure 1:**
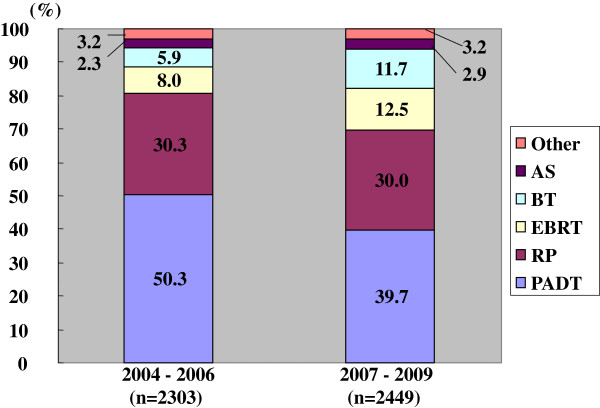
**Distribution of the primary therapy of all 4752 patients (Chi-square test; p < 0.001).** RP: radical prostatectomy, PADT: primary androgen deprivation therapy, EBRT: external beam radiation therapy, BT: brachytherapy, AS: active surveillance.

Half of the patients received PADT in the prior period, while approximately 40% of patients received PADT in the latter period. Combined androgen blockade (CAB) was the method used in 90% of these in the prior and 94% of these in the latter period, respectively. The trend to use CAB was significantly higher in the latter than the prior period (p < 0.001). The proportion of radical prostatectomy (RP) was the same in the two groups. The proportion of radiation therapy (RT), including both external beam radiation therapy (EBRT) and brachytherapy (BT), increased in the latter period. The primary therapy was thus significantly different between the prior and the latter periods (p < 0.001) (Figure 1).

**Figure 2:**
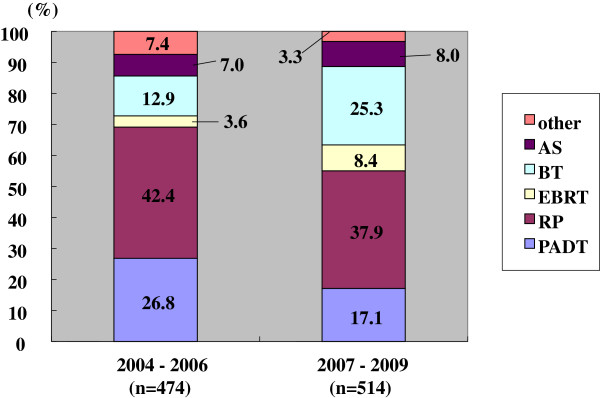
**Distribution of the primary therapy of the low risk patients (Chi-square test; p < 0.001).** RP: radical prostatectomy, PADT: primary androgen deprivation therapy, EBRT: external beam radiation therapy, BT: brachytherapy, AS: active surveillance.

**Figure 3:**
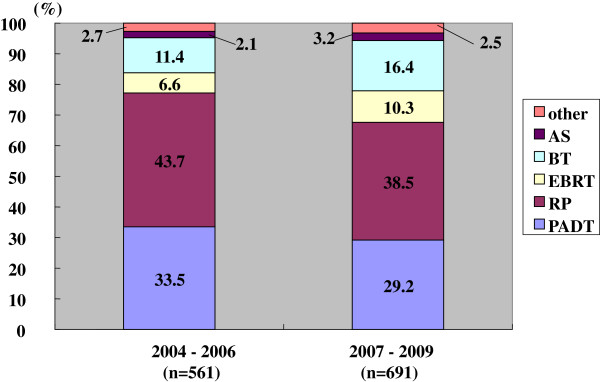
**Distribution of the primary therapy of the intermediate risk patients (Chi-square test; p = 0.013).** RP: radical prostatectomy, PADT: primary androgen deprivation therapy, EBRT: external beam radiation therapy, BT: brachytherapy, AS: active surveillance.

**Figure 4:**
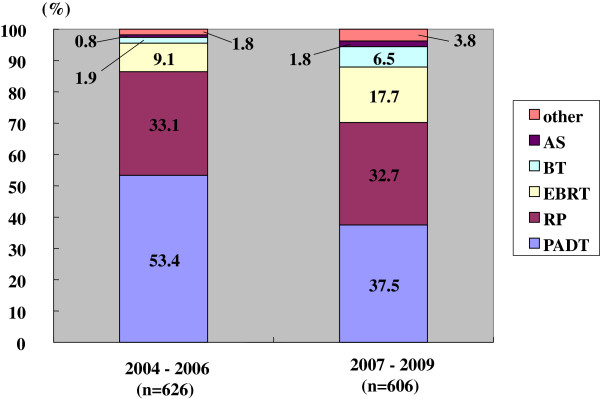
**Distribution of the primary therapy of the high risk patients (Chi-square test; p < 0.001).** RP: radical prostatectomy, PADT: primary androgen deprivation therapy, EBRT: external beam radiation therapy, BT: brachytherapy, AS: active surveillance.

**Figure 5:**
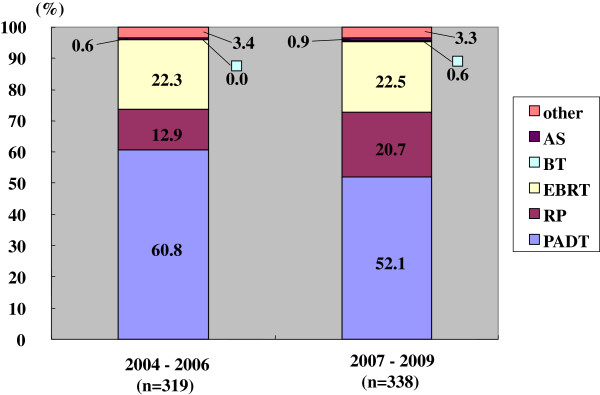
**Distribution of the primary therapy of the very high risk patients (Chi-square test; p = 0.068).** RP: radical prostatectomy, PADT: primary androgen deprivation therapy, EBRT: external beam radiation therapy, BT: brachytherapy, AS: active surveillance.

The primary therapy had thus changed between the prior and the latter periods (Figures 2, 3, 4, 5). The use of PADT decreased significantly. On the other hand, the proportion of RT increased. Such a significant change in primary therapy was seen in the low, intermediate and high risk groups (p < 0.001, p = 0.013, and p < 0.001, respectively). In the very high risk group, this change was marginal (p = 0.068).

## Discussion

We have previously investigated the trend of risk classification and primary therapy in patients who had been diagnosed with prostate cancer in the Nara Uro-oncological Research Group registry between 2004 and 2006 [1]. Half of the patients showed high risk features and received PADT according to this report. This result was compatible with reports by the Japanese Urological Association (JUA) [3,6] that 57% and 50% of patients received PADT in 2000 and 2004, respectively. We concluded that the higher use of PADT and the higher proportion of high risk patients were distinctive trends among Japanese prostate cancer patients compared with those in the USA [2,7,8].

Three years after our first report, we conducted the present study to clarify any changes in the trends of risk classification and primary therapy in the NUORG data registry between 2007 and 2009. We found significant changes both in risk classification and primary therapy. The proportion of patients with a high or greater risk had significantly decreased. On the other hand, the proportion with a low risk remained constant, and that with an intermediate risk increased. The conceivable reason for this migration in the risk classification to an intermediate risk was caused by the significant decrease in high risk patients and the introduction of the new Gleason grading proposed by the 2005 International Society of Urologic Pathology (ISUP) Gleason Grading Consensus [9]. Indeed, the proportion of patients with a higher PSA value at the time of diagnosis had significantly decreased, while the proportion of patients with a Gleason score of 7 had significantly increased (Table 1) in the present study.

The present study did not only reveal a significant shift in the risk classification between the prior and latter periods, but also a higher proportion of patients with a high or greater risk in Japan than in the USA [10,11]. One possible reason for this trend is the difference in the PSA exposure rate between the USA and Japan. The PSA exposure rate was lower in Japan than in the United States [12,13]. In other words, Japanese urologists still have to treat patients with a high or greater risk.

Another aspect of the present study was the change in primary therapy in Japan. Our previous paper showed that half of the patients received PADT between 2004 and 2006, and doctors at hospitals where modalities for radiation therapy were not available usually chose PADT if the patients were unwilling to undergo radical prostatectomy [1,14]. On the other hand, the proportion of PADT significantly decreased from 50% to 40% in the latter period. The proportion of radical prostatectomy had not changed (about 30%), but the proportion of radiation therapy had significantly increased from 14% to 24%. In Japan, low-dose-rate brachytherapy was approved in 2003. Coincidently, intensity modulated radiation therapy (IMRT) has come to be widely used. The excellent oncologic outcome of radiation therapy has been recognized during the last decade [15,16]. These circumstances likely had an influence on the decision concerning primary therapy.

The changes in primary therapy in the low, intermediate, and high risk groups were also significantly different in the prior and latter periods (Figures 2, 3, 4). The use of PADT had significantly decreased and the proportion of radiation therapy had increased, except for cases with a very high risk. However, the proportion of PADT in Japan is still higher than in the USA.

## Conclusion

A significant shift in risk classification toward a lower risk could be seen in Japanese prostate cancer patients between the 2004-2006 and 2007-2009 periods. However, there were still higher risk patients than in the USA. The primary therapy also changed during the 3 years. The use of PADT strongly decreased and the proportion of radiation therapy increased, not only in the overall population, but also in each risk group separately.

## Abbreviations

PADT: Primary androgen deprivation therapy; NUORG: Nara Uro-Oncological Research Group; PSA: Prostate-specific antigen,; RP: Radical prostatectomy; RT: Radiation therapy; EBRT: External beam radiation therapy; BT: Brachytherapy.

## Competing interests

The authors declare that they have no competing interests.

## Authors’ contributions

All authors made substantial contributions to the acquisition and interpretation of data, critical revision of the manuscript for important intellectual content, and approved the final version for publication. KF made substantial contributions to the conception and design of the study. NT performed the statistical analysis.

## Pre-publication history

The pre-publication history for this paper can be accessed here:

http://www.biomedcentral.com/1471-2407/13/588/prepub
